# Human Placental Mesenchymal Stem Cells-Exosomes Alleviate Endothelial Barrier Dysfunction via Cytoskeletal Remodeling through hsa-miR-148a-3p/ROCK1 Pathway

**DOI:** 10.1155/2024/2172632

**Published:** 2024-04-20

**Authors:** Yuzhen Lv, Wenqin Yu, Ruiui Xuan, Yulu Yang, Xiaolan Xue, Xiaowei Ma

**Affiliations:** ^1^School of Clinical, Ningxia Medical University, Yinchuan 750003, China; ^2^Ningxia Institute for Human Stem Cell Research, General Hospital of Ningxia Medical University, Yinchuan 750003, China; ^3^Intensive Care Unit, Cardiocerebral Vascular Disease Hospital, General Hospital of Ningxia Medical University, Yinchuan 750003, China

## Abstract

**Background:**

Endothelial barrier disruption of human pulmonary vascular endothelial cells (HPVECs) is an important pathogenic factor for acute lung injury (ALI)/acute respiratory distress syndrome (ARDS). Mesenchymal stem cells-exosome (MSCs-Exo) represents an ideal carrier for cell-free therapy. The therapeutic implication and underlying mechanism of human placental MSCs-Exo (HPMSCs-Exo) in ALI/ARDS need to be further explored.

**Materials and Methods:**

HPMSCs-Exo was extracted from HPMSCs and characterized. Then, the therapeutic effects of exosomes were evaluated in ALI mice and HPVECs. RNA-sequencing was applied to reveal the miRNA profile of HPMSCs-Exo and differentially expressed genes (DEGs) in HPMSCs-Exo-pretreated HPVECs. The targets of miRNAs were predicted by bioinformatics methods and correlated to DEGs. Finally, the role of hsa-miR-148a-3p/ROCK1 pathway in HPVECs has been further discussed.

**Results:**

The results showed that HPMSCs-Exo could downregulate Rho-associated coiled-coil-containing protein kinase 1 (ROCK1), upregulate the expression of zonula occludens-1 (ZO-1) and F-actin, promote HPVECs migration and tube formation, reduce cytoskeletal disorders and cell permeability, and thus improve ALI/ARDS. RNA-sequencing revealed the DEGs were mainly enriched in cell junction, angiogenesis, inflammation, and energy metabolism. HPMSCs-Exo contains multiple miRNAs which are associated with cytoskeletal function; the expression abundance of hsa-miR-148a-3p is the highest. Bioinformatic analysis identified ROCK1 as a target of hsa-miR-148a-3p. The overexpression of hsa-miR-148a-3p in HPMSCs-Exo promoted the migration and tube formation of HPVECs and reduced ROCK1 expression. However, the overexpression of ROCK1 on HPVECs reduced the therapeutic effect of HPMSCs-Exo.

**Conclusions:**

HPMSCs-Exo represents a protective regimen against endothelial barrier disruption of HPVECs in ALI/ARDS, and the hsa-miR-148a-3p/ROCK1 pathway plays an important role in this therapeutics implication.

## 1. Introduction

Acute lung injury (ALI)/acute respiratory distress syndrome (ARDS) is a progressive, hypoxic respiratory failure caused by a variety of pathogenic factors both within and outside the lungs, in which its pathogenesis has not been fully elucidated, and it was difficult to treat clinically [1–3]. An important study on ARDS reported that the unadjusted hospital mortality rate was 35%, 40%, and 46% among those with mild, moderate, and severe ARDS, respectively [4]. A previous study suggested that impaired lung endothelial barrier function may lead to increased permeability, which is key to the development of lung injury [5]. Human placental vascular endothelial cells (HPVECs) are an important component of the lung barrier and play a decisive role in angiogenesis, cell migration, and permeability, and their proper function depends on the integrity of the cytoskeleton [6, 7]. Therefore, effectively improving the function of HPVECs and reducing permeability are of great significance for ALI/ARDS.

Cellular therapy is considered a promising approach to treat organ dysfunction. Several animal studies have demonstrated the therapeutic effects of mesenchymal stem cells (MSCs) [8]. However, stem cell therapy also has side effects that are worthy of further assessment [9]. As research has progressed, it has been found that MSCs-exosomes (MSCs-Exo) have similar functions to the parent cells, which are considered a hot research topic for cell-free therapies [10]. Exosomes are the smallest type of extracellular vesicles, with a diammer of phospholipid bilayers ranging from 30 to 150 nm [11]. Due to their properties, MSCs-derived exosomes have demonstrated a remarkable ability to provide therapeutic benefits to a certain extent. They can be used as carriers to transport the tiny ribonucleic acids (microRNAs (miRNAs)) to recipient cells [10, 12]. Studies have shown that miRNAs are involved in vascular endothelial cell differentiation, proliferation, apoptosis, and catabolic processes, and they play an essential role in ALI/ARDS [13]. MiRNAs can control the expression levels of different cytoskeletal molecules and upstream regulatory signaling pathways. Therefore, they play a more critical role in the biochemical pathways of normal cell shape, movement, and contraction [14]. However, their application in cell-free therapy still requires further research.

Although various MSCs are widely used in research, the characteristics of the different sources and their miRNAs are different [15]. The placenta is a transient organ that maintains fetal tolerance and constitutes a rich reservoir of MSCs [16, 17]. Compared with MSCs from other sources, human placental MSCs (HPMSCs) can be easily isolated from the placenta. Their derived cells are less affected by age and environmental factors, do not require invasive procedures, are not harmful to fetuses and mothers, and do not cause ethical issues that have received widespread attention [18]. HPMSCs are rich in sources and easily accessible, while there is still a lack of systematic integration of miRNA species. In the present study, HPMSCs were selected as donor cells to explore the underlying mechanisms of exosomes. We explored the influences of exosomes on the barrier function of HPVECs from the functional experiments and genetic assays. We also detected the miRNA expression profiles of exosomes by high-throughput sequencing.

## 2. Materials and Methods

### 2.1. Induced Differentiation of HPMSCs

The fourth-generation HPMSCs were obtained from the Institute of Human Stem Cell Research at the General Hospital of Ningxia Medical University (Yinchuan, China). The cells were inoculated in 12-well plates (3513; Corning Inc., Corning, NY, USA), including three induction groups and one control group. After the cells reached a confluence of 80%–90%, induction differentiation media (05412, 05455, and 05465; STEMCELL Technologies, Vancouver, Canada) were added to the positive wells, and the media were changed every 2–3 days to observe the morphological changes of the cells under a microscope (CKX41; Olympus, Tokyo, Japan). After 21 days, the cells were stained with different staining methods.

### 2.2. Cell Culture

Here, 2 × 10^5^ HPMSCs were cultured and then incubated in a 5% carbon dioxide incubator (Thermo Fisher Scientific, Waltham, MA, USA) at 37°C for 7 days, followed by digestion in a petri dish every 24 hr, cell counting, and drawing the growth curve.

### 2.3. Animal Feeding

Male C57BL/6 mice aged 6–8 weeks were provided by Ningxia Medical University Animal Center. Mice were raised in a specific pathogen-free grade animal room with 40%–60% humidity and maintained in a 12 hr light/dark cycle. The mice had free access to food and water.

### 2.4. Extraction and Identification of HPMSCs-Exo

HPMSCs were resuscitated and cultured in a serum-free medium (X-VIVO-15, Lonza Inc., Basel, Switzerland). The culture supernatant was collected and centrifuged at 3,000x *g* for 15 min (Eppendorf, Hamburg, Germany) to remove the cell debris. The centrifuged supernatant was transferred to a new centrifuge tube and operated according to the instructions of the SeraMir Exosome RNA Purification Kit (RA806A-1, SBI, Palo Alto, CA, USA). The cell morphology was observed under a transmission electron microscope (H7650, Hitachi, Tokyo, Japan), and surface markers (CD9 and CD63; Proteintech, Chicago, IL, USA) were identified by western blot analysis.

### 2.5. Uptake of HPMSCs-Exo

A Dil red fluorescent probe (C1036; Beyotime Institute of Biotechnology, Shanghai, China), a Dio green fluorescent probe (C1038, Beyotime Institute of Biotechnology), and a DIPA blue fluorescent dye (C0065; Beijing Solarbio Science & Technology Co., Ltd., Beijing, China) were used to label HPMSC-Exo, HPVECs, and the nucleus, respectively. HPMSCs-Exo were cocultured with HPVECs to observe uptake under a fluorescence microscope.

### 2.6. Grouping and Intervention

HPVECs: HPVECs (ScienCell Research Laboratories Inc., Carlsbad, CA, USA) were cultured on an endothelial cell medium (ECM) (1001, ScienCell Research Laboratories Inc.) and incubated in an incubator at 37°C and 5% CO_2_. The cells were grouped into normal control (NC) group, HPMSCs-Exo group (100 *µ*g/mL), lipopolysaccharide (LPS; O11:B6; Sigma–Aldrich, St. Louis, MO, USA)-treated damage group (100 ng/mL, LPS group), and HPMSCs-Exo treatment group (HPMSCs-Exo + LPS group). Except for the NC and HPMSCs-Exo groups, HPVECs in the other two groups were treated with LPS, and HPMSCs-Exo were added to the HPMSCs-Exo + LPS group for a 24-hr coculture.

Mice: Thirty-two 6-week-old healthy C57BL/6 male mice were randomly divided into four groups: NC group (30 *μ*L saline by tracheal drip), HPMSCs-Exo group (30 *μ*L exosome suspension by tracheal drip), lipopolysaccharide-treated damage group (LPS at 2.5 mg/kg dissolved in 30 *μ*L saline), and HPMSCs-Exo + LPS group (30 *μ*L HPMSCs-Exo suspension by tracheal drip after 12 hr in the LPS group).

### 2.7. Cell Migration Assay

Here, 1 × 10^4^ HPVECs were inoculated into a 0.8 mm Transwell chamber (24 holes), and the culture medium containing 15% fetal bovine serum (FBS) was added into the lower chamber to induce cell migration. After 8 hr, the culture medium was discarded, fixed with 4% paraformaldehyde solution (P1110; Beijing Solarbio Science & Technology Co., Ltd.) for 30 min, thrice washed with phosphate-buffered saline (PBS), stained with 1% crystal violet solution (C0121; Beyotime Institute of Biotechnology) for 8 min, washed with PBS for three times, and observed, and images were captured under a microscope.

### 2.8. Tube Formation Assay

Here, 50 *µ*L of growth factor-reduced Matrigel (Becton Dickinson) was dispensed into 96-well tissue culture plates at 4°C, and the process was performed on ice to prevent solidification. HPVECs (10^4^ cells/well) were seeded onto Matrigel-coated 96-well plates. The tube formation of endothelial cells was observed within 6–8 hr, and photos were taken under a microscope.

### 2.9. Phalloidin Cytoskeleton Staining

HPVECs were cultured and twice washed with PBS (70011-44; Gibco, New York, NY, USA). The cells were then fixed with 4% paraformaldehyde solution at room temperature for 30 min. Cells were twice washed with PBS, each time for 10 min. The cells were permeabilized with 0.5% Triton X-100 (9002-93-1; China) for 5 min. Cells were twice washed with PBS, each time for 10 min. Take the proper amount of phalloidin fluorescein conjugate working solution (B7678; APEBIO Inc., Carlsbad, CA, USA), cover the cells, incubate the cells for 30 min at room temperature in the dark, and then, thrice wash the cover glass with PBS, each time for 5 min. Finally, the 4′,6-diamidino-2-phenylindole (DAPI) solution was added to restain the nucleus for about 30 s. The cells were observed under a fluorescence microscope or a confocal microscope.

### 2.10. Histological Analysis

The left lobe of lung tissue was fixed in paraformaldehyde solution for 24 hr. After dehydration and transparency, the slices of 5 *μ*m were made by routine paraffin embedding. The pathological changes in lung tissue were observed by hematoxylin and eosin staining, dehydration, and sealing.

### 2.11. Lung Wet/Dry Ratio

The right lung tissue of mice was weighed on the electronic balance, and the lung wet mass (wet mass, *W*) was recorded. The lung wet mass (dry mass, *D*) was dried in a constant temperature oven at 65°C and then weighed again, and the lung dry mass (lung dry mass) was recorded. The *W*/*D* ratio of lung tissue was calculated.

### 2.12. Western Blot Analysis

RIPA buffer (KGP2100; KeyGen Biotech, Nanjing, China) was used to lyse cells, and the extracted proteins were quantified via BCA assay. A total of 30 *µ*g of each sample was then separated using sodium dodecyl-sulfate polyacrylamide gel electrophoresis (SDS–PAGE; 90 min, 120 V, 60 mA), followed by transferring onto polyvinylidene difluoride (PVDF) membranes (Merck Millipore, Darmstadt, Germany). Blots were blocked with 5% nonfat milk and incubated overnight with appropriate primary antibodies at 4°C, and glyceraldehyde 3-phosphate dehydrogenase (GAPDH) served as a loading control. Afterwards, the membranes were incubated with primary antibodies (RHOA (10749-1-AP; Proteintech), ROCK1 (21850-1-AP; Proteintech), VE-cadherin (ab232880; Abcam, Cambridge, UK), ZO-1 (ab276131; Abcam)) at 4°C overnight. Blots were then probed with the secondary horseradish peroxidase (HRP)-conjugated anti-rabbit IgG antibody (Proteintech) for 1 hr at room temperature. Protein bands were subsequently detected using the SignalFire™ ECL Reagent, and the ImageLab software (ver. 4.1; Bio-Rad Laboratories Inc., Hercules, CA, USA) was used for the subsequent data analysis.

### 2.13. The Mitochondrial Membrane Potential (MMP)

HPVECs in different groups were detected with JC-1 staining solution (C2006; Beyotime Institute of Biotechnology). After the culture medium was discarded, the cells were twice washed with PBS, and JC-1 staining solution was added and incubated at 37°C for 20 min. After incubation, the supernatant was removed, the cells were twice washed with JC-1 staining solution (1×), and the cells were added to the medium and observed under a fluorescence microscope.

### 2.14. RNA Sequencing (RNA-Seq) Analysis

HPVECs and HPMSCs-Exo were sent to BioMarker company for RNA-Seq. First, exosomal miRNAs were assessed to ensure their qualification for further analysis. After that, a database was developed, and sequencing was performed on the Illumina platform. The data were analyzed using the BMKCloud platform (https://www.biocloud.net). Gene or transcript expression levels were quantified using fragments per kilobase of transcript per million fragments mapped (FPKM), and functional enrichment analysis of differentially expressed genes (DEGs) was performed using “edgeR” package in R software, which could be used with or without biological replicates. The fold-change (FC) represents the ratio of the expression volume in the two samples (groups), and the false discovery rate (FDR) was defined as the ratio of the number of false-positive results to the number of total positive test results. In the present study, FC > 1.5 and *P* value < 0.05 were selected as the screening criteria. DEGs were further analyzed by the Gene Ontology (GO) (http://www.geneontology.org) and the Kyoto Encyclopedia of Genes and Genomes (KEGG) (http://www.genome.jp/kegg/) pathway enrichment analyses to determine functional and biological properties. In addition, using hypergeometric distribution, enrichment analysis was carried out on the pathway.

### 2.15. Quantitative Polymerase Chain Reaction (q-PCR)

Total RNA was extracted from cells using TRIzol reagent (15596026; Invitrogen, Carlsbad, CA, USA). The q-PCR was employed to detect the expression levels of DEGs. Total RNA was extracted from each sample. GAPDH or U6 were used as internal reference genes. The 2^−*ΔΔ*Ct^ method was used to calculate the relative expression level of the target gene. The q-PCR was performed using miRCURY SYBR Green PCR Kit (Exiqon–Qiagen) according to the manufacturer's instructions with StepOnePlus Real-Time PCR System (ABI, Thermo Fisher Scientific). Each experiment was repeated at least three times. The primers used for the q-PCR are listed in Table 1.

### 2.16. Transfection

HPMSCs were seeded at a density of 2 × 10^5^ cells/well. When the HPMSCs reached a confluence of 70%, they were transfected with hsa-miR-148a-3p mimic or NC mimic (Gene JiKai Co. Ltd., Shanghai, China), using the Lipofectamine 2000 (Invitrogen) according to the manufacturer's instructions.

### 2.17. Statistical Analysis

The data were presented as mean ± standard deviation (SD). Two-way analysis of variance (ANOVA) was applied to compare differences between treatment groups. *P*  < 0.05 was considered statistically significant.

## 3. Results

### 3.1. Characteristics of HPMSCs and HPMSCs-Exo

**Figure 1:**
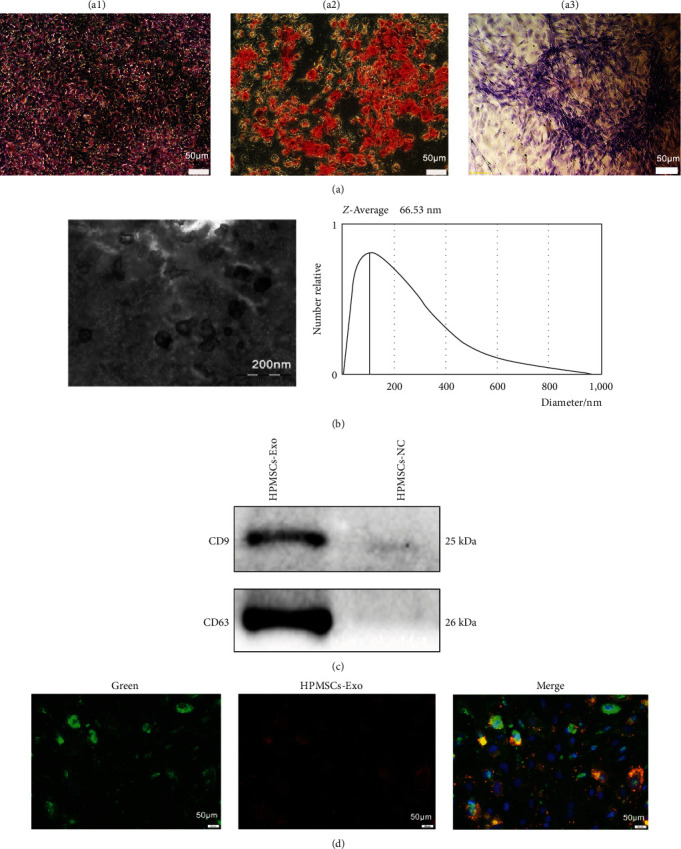
Characteristics of the HPMSCs and HPMSCs-Exo. (a) Induced differentiation of HPMSCs under a microscope ((a1) adipogenic differentiation, (a2) osteogenic differentiation, (a3) chondrogenic differentiation. Scale bar = 50 *μ*m). (b) Morphological observation of HPMSCs-Exo under EM (scale bar = 200 nm). (c) Identification of surface markers of HPMSCs-Exo. (d) Uptake of HPMSCs-Exo by HPVECs. (Scale bar = 50 *μ*m).

The differentiation of HPMSCs into adipocytes, chondrocytes, and osteocytes was examined. At 21 days post-induction, adipogenic differentiation cells were filled with purplish red lipid droplets after staining with oil red O, osteogenic differentiation cells were observed as pale red calcium-like tissue deposits after staining with Alizarin red, and chondrogenic differentiation cells appeared as blue cartilage clusters after staining with toluidine blue (Figure 1(a)). HPMSCs-Exo were circular or oval under EM, with a dual-membrane and diameter of <150 nm (Figure 1(b)). Western blotting showed positive expression of the surface markers (CD9 and CD63), which is consistent with the properties of the exosomes (Figure 1(c)). Labeling HPMSCs-Exo and HPVECs by different fluorescent dyes that were harmless to the cell survival showed that HPMSCs-Exo could be successfully ingested by HPVECs (Figure 1(d)).

### 3.2. The Effects of HPMSCs-Exo on Lung-Injured Mice

**Figure 2:**
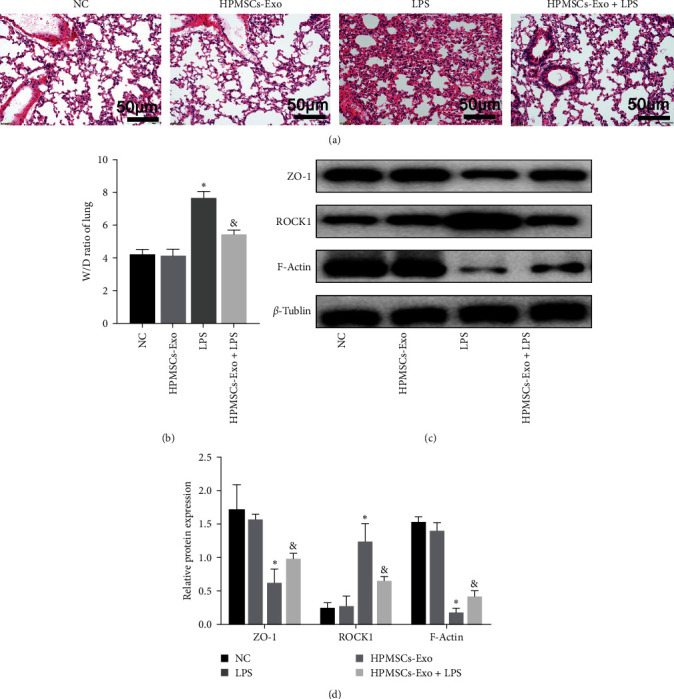
Effects of HPMSCs-Exo on lung-injured mice. (a) Representative hematoxylin- and eosin-stained sections of lung tissue (scale bar = 50 *μ*m). (b) The values of lung *W*/*D* of each group. (c) Immunoblot analysis of permeability-related proteins and cytoskeleton-related proteins. (d) Quantification of the immunoblots, in which the results were expressed as the mean ± SD of at least three experiments. The results showed that HPMSCs-Exo increased the expression levels of F-actin and ZO-1, while it decreased the expression levels of ROCK1 on lung-injured mice.  ^*∗*^*P* < 0.05 vs. NC, ^&^*P* < 0.05 vs. LPS.

We assayed the changes of lung histopathology in each group. As shown in Figure 2(a), we can see that the structure of lung lobules in NC group and HPMSCs-Exo was intact and has normal alveolar wall and no inflammatory cell infiltration. In LPS group, there was a large number of infiltrated inflammatory cells in lung tissue, the alveolar septum became thicker, the alveolar structure was destroyed, and bleeding focus was found. In HPMSCs-Exo + LPS group, interventions of HPMSCs-Exo could remarkably alleviate the pathological damage of lung in ALI mice (Figure 2(a)). In order to detect the effect of HPMSCs-Exo on pulmonary edema, we assayed the lung (*W*/*D*) ratio. Compared with the NC group and HPMSCs-Exo group, the *W*/*D* ratio in LPS group was significantly increased (Figure 2(b)). Compared with the LPS group, HPMSCs-Exo + LPS groups dramatically reduced the elevated *W*/*D* ratio. The results displayed that HPMSCs-Exo can relieve the degree of pulmonary edema in ALI mice.

ZO-1 and F-actin can ensure the integrity of the cytoskeleton and permeability [19]. Immunoblot analysis of the ZO-1 and F-actin showed that their expression levels decreased in the LPS group, while those increased after HPMSCs-Exo treatment. ROCK1 is involved in the regulation of the cytoskeleton [20]. Immunoblotting of ROCK1 revealed that HPMSCs-Exo could reduce protein expression levels to improve the cytoskeleton by inhibiting the activity (Figures 2(c) and 2(d)).

### 3.3. The Effects of HPMSCs-Exo on Tube Formation and Migration Capabilities of HPVECs

**Figure 3:**
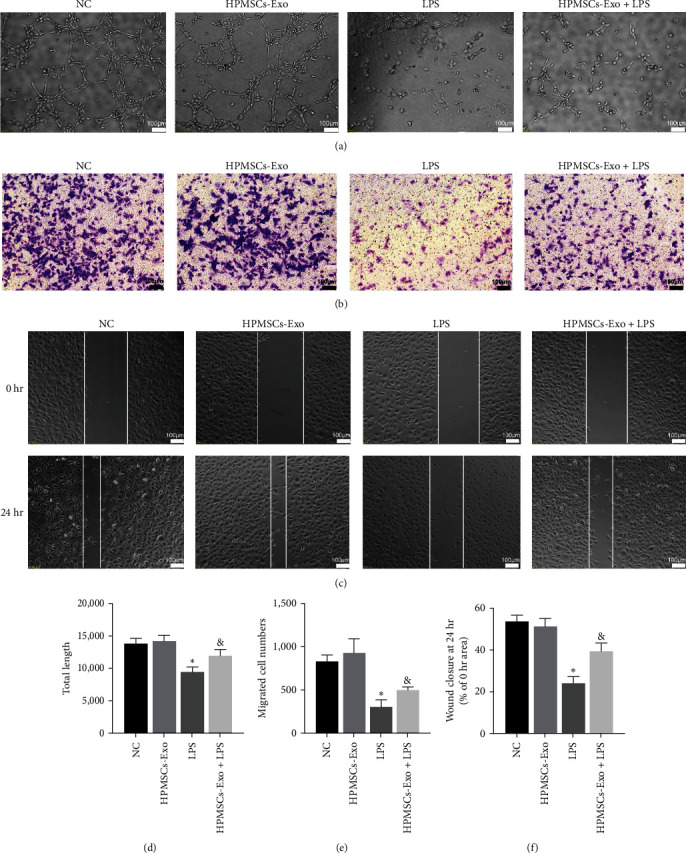
The effects of HPMSCs-Exo on tube formation and migration capabilities of HPVECs. (a) The representative images of tube formation capability of HPVECs. (d) The average length from a randomly selected region was quantified (*n* > 3). The results showed that HPMSCs-Exo reversed the decline of tube formation capability of LPS-treated HPVECs (scale bar = 100 *μ*m). (b) The representative Giemsa staining images of transwell assay of HPVECs. (e) The average number of migrated cells from a randomly selected region was counted (*n* > 3). The results showed that HPMSCs-Exo reversed the decrease of migration capability of LPS-treated HPVECs (scale bar = 100 *μ*m). (c) The representative images of the migration of HPVECs, with lines approximating the cell front. (f) The average quantification of the mobility ((0−24 hr)/24 hr% area) from a randomly selected region was calculated (*n* > 3). The results showed that HPMSCs-Exo could improve the migration capability of LPS-insulted HPVECs.  ^*∗*^*P* < 0.05 vs. NC, ^&^*P* < 0.05 vs. LPS.

The effects of HPMSCs-Exo on the angiogenesis of HPVECs were examined by tube formation assay. Compared with NC and HPMSCs-Exo groups, the tube formation of HPVECs was significantly inhibited in the LPS group. After HPMSCs-Exo treatment, it was found that the tube formation capability was recovered (Figures 3(a) and 3(d)). In addition, the transwell assay was used to detect the effects of HPMSCs-Exo on the migration capability of HPVECs. The results showed that the migration capability decreased in the LPS group, while it increased in the HPMSCs-Exo + LPS group (Figures 3(b) and 3(e)). The wound healing assay was employed to further evaluate the cell migration capability (Figures 3(c) and 3(f)), which was consistent with the results of the transwell assay. The results indicated that HPMSCs-Exo could promote the migration and vascularization capabilities of HPVECs.

### 3.4. The Effects of HPMSCs-Exo on Cytoskeletal Changes and MMP of HPVECs

**Figure 4:**
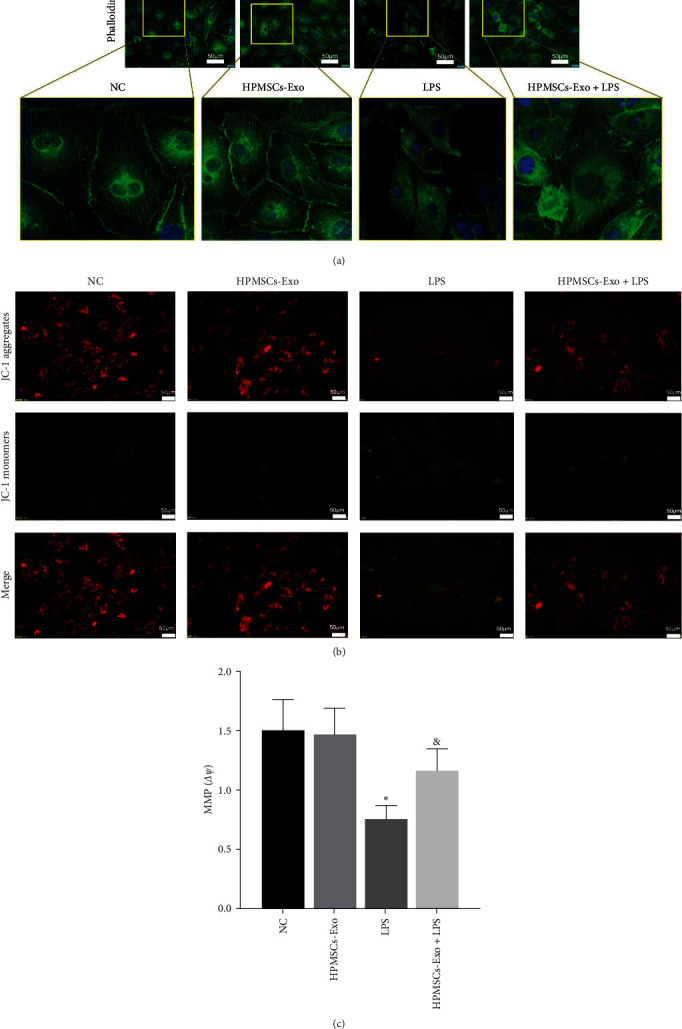
Cytoskeletal changes and mitochondrial function of HPVECs. (a) Phalloidin staining of cytoskeleton of HPVECs. HPMSCs-Exo promoted cytoskeletal rearrangements (scale bar = 20 *μ*m). (b) MMP of HPVECs. The red fluorescence represents the JC-1 aggregates, the green fluorescence represents the monomers, and the red–green fluorescence ratio (*ΔΨ*) is used for the MMP (scale bar = 50 *μ*m). (c) MMP-related fluorescence intensity. ^*∗*^*P* < 0.05 vs. NC, ^&^*P* < 0.05 vs. LPS.

Cytoskeleton damage to endothelial cells can lead to abnormalities in morphology, migration, angiogenesis, and permeability and eventually lead to apoptosis [21]. Phalloidin staining was used to observe changes in the cytoskeleton of HPVECs, which showed clear cell outlines, well-defined cell junctions, and well-arranged cytoskeletal proteins in the NC and HPMSCs-Exo groups. In contrast, after stimulation with LPS, the HPVECs were ill-defined, and the cytoskeleton proteins were disordered. After HPMSCs-Exo treatment, the cell morphology was acceptable, and cytoskeletal protein disorder was improved. Although the arrangement of the cytoskeleton proteins was slightly disordered compared with the NC group, the structure of the cytoskeleton proteins could still be identified (Figure 4(a)). The detection of MMP not only indicated cellular mitochondrial function but also the occurrence of apoptosis [22]. As shown in Figure 4(b), bright red and weak green fluorescence could be observed in NC and HPMSCs-Exo groups. After being treated with LPS, there was a decrease in the intracellular red fluorescence intensity, while green fluorescence was significantly enhanced, indicating the decreased MMP of HPVECs, while it could be reversed after HPMSCs-Exo treatment (Figure 4(c)).

### 3.5. RNA-Seq and GO and KEGG Pathway Enrichment Analyses

**Figure 5:**
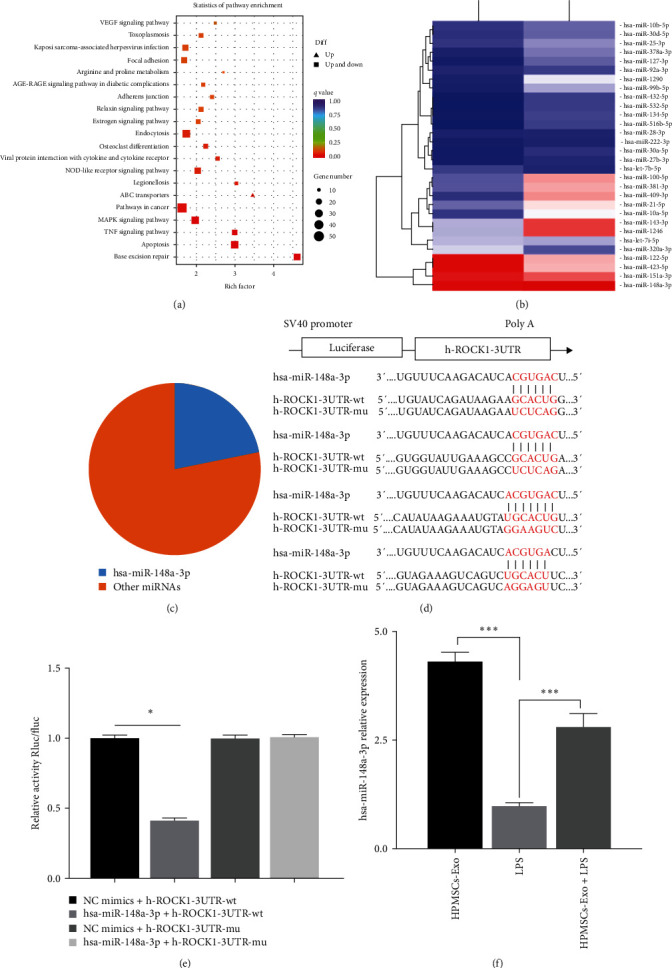
Results of RNA-seq of HPVECs. (a) KEEG pathway analysis of the DEGs in the HPVECs. The top 20 pathways with the most reliable significant enrichment were selected. Each circle represents a KEGG path; the ordinate representing the path name and the abscissa is the enrichment factor representing the proportion of genes in a differential gene annotated to a pathway to all genes. The circle color *q* value is the corrected *P*-value; the size of the circle indicates the number of genes enriched in the pathway. The path was represented by the graph near the bottom right corner (the larger the reference value). (b) The miRNA expression levels of HPMSCs-Exo (top 30). (c) The proportion of hsa-miR-148a-3p in HPMSCs-Exo miRNA profiles. (d) ROCK1 gene is the downstream target gene of hsa-miR-148a-3p. (e) Luciferase reporter assays proved that hsa-miR-148a-3p can bind to h-ROCK1-3UTR directly.  ^*∗*^*P* < 0.05. (f) HPMSCs-Exo can increase the expression level of hsa-miR-148a-3p in the LPS-insulted HPVECs.  ^*∗∗∗*^*P* < 0.01.

To further investigate the effects of HPMSCs-Exo on LPS-treated HPVECs, we detected the DEGs in LPS and HPMSCs-Exo + LPS groups, and it was found that DEGs were analyzed by the KEEG pathway analysis, in which up to 20 pathways of differential activation were selected (Figure 5(a)). These pathways were mainly enriched in vascular endothelial growth factor (VEGF) signaling pathway, adhere junction, inflammation-related pathways (tumor necrosis factor (TNF) and mitogen-activated protein kinase (MAPK) signaling pathways), and disease-related pathways.

Exosomal miRNAs have diverse functions, such as participation in inflammatory reactions, cell migration, proliferation, apoptosis, and autophagy [23]. We explored miRNA profiles of HPMSCs-Exo and listed the top 30 miRNAs (Figure 5(b)). Among them, the hsa-miR-148a-3p expression level was the highest (Figure 5(c)). It was biologically revealed that ROCK1 gene is the downstream target gene of hsa-miR-148a-3p (Figure 5(d)). Luciferase reporter assays proved that hsa-miR-148a-3p could bind to h-ROCK1−3UTR directly (Figure 5(e)). Besides, the hsa-miR-148a-3p expression level was compared between LPS and HPMSCs-Exo + LPS groups, and the results showed that HPMSCs-Exo could increase hsa-miR-148a-3p expression level (Figure 5(f)). Binding to HPMSCs-Exo could reduce the ROCK1 expression level (Figure 2(c)), which confirmed that HPMSCs-Exo hsa-miR-148a-3p/ROCK1 pathway could play an important role in the adjustment in mice with acute lung injury and HPVECs.

### 3.6. The Effects of HPMSCs-Exo on HPVECs via hsa-miR-148a-3p

**Figure 6:**
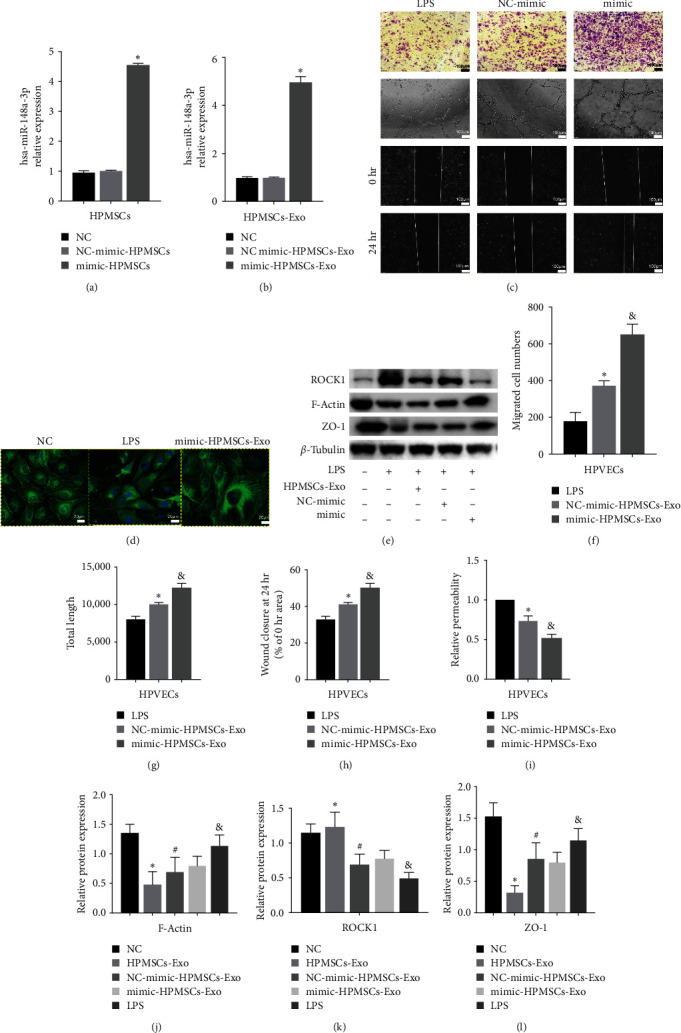
The effects of mimic-HPMSCs-Exo on the function of HPVECs. (a and b) Overexpression of hsa-miR-148a-3p in both HPMSCs and HPMSCs-Exo was determined by q-PCR.  ^*∗*^*P* < 0.05 vs. NC. (c) Representative Giemsa staining and tube-forming and migration capacity image of mimic-HPMSCs-Exo acting on HPVECs (scale bar = 100 *μ*m). (f, g, and h) The number of migrating cells in randomly selected regions (*n* > 3). The results showed that mimic-HPMSCs-Exo had significantly promoted the migration of HPVECs. The mean length quantification of statistically randomly selected regions (*n* > 3). The results showed that mimic-HPMSCs-Exo had the strongest ability to promote angiogenesis of HPVECs. The average mobility from randomly selected regions was quantified (*n* > 3). The results showed that HPMSCs-Exo had the strongest ability to promote wound healing of HPVECs.  ^*∗*^*P* < 0.05 vs. LPS, ^*&*^*P* < 0.05 vs. NC-mimic-HPMSCs-Exo. (d) Phalloidin staining of cytoskeleton of HPVECs (scale bar = 20 *μ*m). (i) HPMSCs seeded on transwell plates were acted with mimic-HPMSCs-Exo, and FITC-dextran relative fluorescence intensity was determined (*n* > 3).  ^*∗*^*P* < 0.05 vs. LPS, ^*&*^*P* < 0.05 vs. NC-mimic-HPMSCs-Exo. (e) Immunoblot analysis of ROCK1 and F-actin expression level in HPVECs. (j, k, and l) Quantification of the immunoblots, in which the results were expressed as the mean ± SD of at least three experiments.  ^*∗*^*P* < 0.05 vs. NC, ^#^*P* < 0.05 vs. LPS, ^&^*P* < 0.05 vs. HPMSCs-Exo.

In the present study, we used the highest miRNA level in HPMSCs-Exo for further analysis. First, mimic-hsa-miR-148a-3p was transfected into HPMSCs to obtain hsa-miR-148a-3p-enriched HPMSCs-Exo (Figures 6(a) and 6(b)). After coculture with HPVECs, mimic-hsa-miR-148a-3p HPMSCs-Exo were found to better promote cell angiogenesis and migration compared with NC-mimic-hsa-miR-148a-3p (Figures 6(c) and 6(f–h)). The effects of hsa-miR148a-3p on HPVECs were further confirmed. Phalloidin staining showed that compared with the LPS group, cell fibers in HPVECs after NC-mimic-hsa-miR-148a-3p treatment were partially existed, and the cell volume increased; after mimic-hsa-miR-148a-3p treatment, the number and thickness of cell fibers in HPVECs increased (Figure 6(d)). Western blotting showed that the ROCK1 expression level further decreased and F-actin expression level further increased after mimic-hsa-miR-148a-3p treatment (Figures 6(e) and 6(j–l)), suggesting that hsa-miR-148a-3p-enriched exosomes could further improve cytoskeleton and alleviate cell damage by reducing ROCK1 expression level in cells. We also used FITC-dextran to determine barrier-protective effects of hsa-miR-148a-3p mimic on LPS-stimulated EC monolayer (Figure 6(i)).

### 3.7. HPMSCs-Exo Promotes Lung Injury Repair through the hsa-miR-148a-3p/ROCK1 Pathway

**Figure 7:**
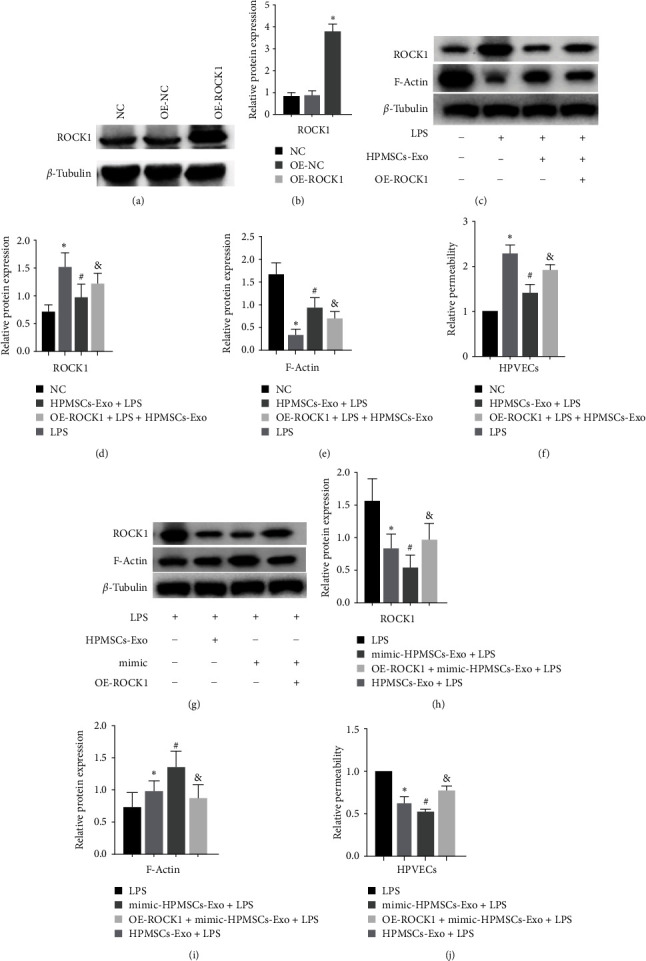
hsa-miR-148a-3p promoted HPVECs repair via targeting ROCK1. (a) Immunoblot analysis of the overexpression of ROCK1 in HPVECs. (b) Quantification of the immunoblots, in which the results were expressed as the mean ± SD of at least three experiments.  ^*∗*^*P* < 0.05 vs. NC. (c) Immunoblot analysis of HPVECs overexpressing ROCK1 by HPMSCs-Exo intervention. (d and e) Quantification of the immunoblots, in which the results were expressed as the mean ± SD of at least three experiments.  ^*∗*^*P* < 0.05 vs. NC, ^#^*P* < 0.05 vs. LPS, ^*&*^*P* < 0.05 vs. HPMSCs-Exo + LPS. (f) FITC-dextran relative fluorescence intensity was determined of HPVECs overexpressing ROCK1 by HPMSCs-Exo intervention, in which the results were expressed as the mean ± SD of at least three experiments.  ^*∗*^*P* < 0.05 vs. NC, ^#^*P* < 0.05 vs. LPS, ^*&*^*P* < 0.05 vs. HPMSCs-Exo+LPS. (g) Immunoblot analysis of HPVECs overexpressing ROCK1 by mimic-HPMSCs-Exo intervention. (h and i) Quantification of the immunoblots, in which the results were expressed as the mean ± SD of at least three experiments.  ^*∗*^*P* < 0.05 vs. LPS, ^#^*P* < 0.05 vs. LPS, ^*&*^*P* < 0.05 vs. mimic-HPMSCs-Exo+LPS. (j) FITC-dextran relative fluorescence intensity was determined of HPVECs overexpressing ROCK1 by mimic-HPMSCs-Exo intervention, in which the results were expressed as the mean ± SD of at least three experiments.  ^*∗*^*P* < 0.05 vs. LPS, ^#^*P* < 0.05 vs. LPS, ^*&*^*P* < 0.05 vs. mimic-HPMSCs-Exo + LPS.

ROCK1 was predicted as a potential target of hsa-miR-148a-3p (Figure 5(d)). The downregulation of ROCK1 has proven to protect the cytoskeleton and permeability, which plays a vital role in lung injury. Therefore, we speculated that the exosomal hsa-miR-148a-3p functioned by downregulating the expression of ROCK1. The overexpression of ROCK1 in endothelial cells (Figures 7(a) and 7(b)) revealed attenuated therapeutic effect of exosomes on lipopolysaccharide-treated cells (Figure 7(c)–7(e)). Using hsa-miR-148a-3p-enriched exosomes to intervene in HPVECs, it was found that the therapeutic effect was further enhanced compared with normal exosomes, but the therapeutic effect in ROCK1-overexpressing cells was weakened (Figure 7(g)–7(i)). The relative fluorescence intensity of different groups was compared by FITC-dextran, and the stronger the fluorescence intensity, the greater the permeability (Figures 7(f) and 7(j)), which further suggest that exosome-derived hsa-miR-148a-3p plays an important role in lung injury by targeting ROCK1.

## 4. Discussion

ALI/ARDS is a devastating disease with a high mortality rate and has become a major public health challenge worldwide, while no effective treatment for this disease has yet been presented [24]. The majority of patients with ALI/ARDS have non-negligible long-term sequelae, including cognitive impairment, residual pulmonary fibrosis, neuromuscular weakness, and persistent neuropathy [25]. HPVECs consist of an ordered layer of flattened squamous cells that reside in the inner layer of the vessel wall and act as a barrier between blood and tissue to ensure basal permeability of the vessel wall through autonomous contraction and connecting structures [26, 27]. Depolymerization of intercellular junctions and cytoskeletal protein remodeling cause structural changes in the HPVECs, which are considered as an important pathogenic factors in alleviating ALI/ARDS [28]. Therefore, maintaining the normal structure of the cytoskeleton and ensuring the normal permeability of HPVECs for ALI/ARDS are worthy of further assessment.

MSCs-Exo are the most popular carriers in cell-free therapy and have therapeutic potential for various injured organs [29]. HPMSCs are excellent donor cells due to their unique advantages [30]. In the present study, HPMSCs were isolated by enzymatic digestion and cultured in the serum-free system. Similar to MSCs from other sources, HPMSCs grew in long spindle-shaped adherence and had the ability to differentiate into adipocytes, osteocytes, and chondrocytes. HPMSCs-Exo was extracted from the culture supernatant, and the electron microscopy showed that they were circular bilayer membranes with a diameter of lower than 150 nm. Western blotting showed that the surface markers (CD9 and CD63) were positive. Exosomes can be taken up by cells to play some functions [31]. In the experiments, red fluorescently labeled HPMSCs-Exo were found around the nucleus of HPVECs under fluorescence microscopy. Migration and angiogenesis are important functions of endothelial cells [32, 33]. Multiple studies have shown that exosomes can affect cell migration and angiogenesis [34], which is consistent with our findings. The permeability of HPVECs is closely associated with cytoskeletal integrity and intercellular junctions [35]. Phalloidin staining showed that the endothelial cell cytoskeleton was complete and well-defined, while after LPS administration, the cytoskeleton was gradually disorganized and lost its intrinsic morphology. Besides, HPMSCs-Exo could promote cytoskeletal integrity. F-actin is an important component of the cytoskeleton in various cell types and is closely associated with the ROCK1 pathway [36], and ZO-1 is the main component of intercellular junctions [21]. In ALI mice, western blot analysis of lung tissue showed that HPMSCs-Exo affected permeability and improved pulmonary edema to some extent by affecting the expression levels of ROCK1 and F-actin. The mitochondria generate most of the chemical energy needed to power the biochemical reactions of cells, and MMP is closely related to apoptosis. The results of the present study showed that HPMSCs-Exo could reverse the decrease in MMP of LPS-treated HPVECs. These results demonstrated that exosomes have multiple protective effects on HPVECs in vivo and in vitro, including improving cell migration and angiogenesis, protecting the cytoskeleton, and promoting autophagy, indicating the therapeutic potential of exosomes in ALI/ARDS.

Exosomal function depends on internal miRNAs [37, 38], playing an important role by binding to target cell genes [39]. We explored the influences of HPMSCs-Exo on the expression levels of DEGs in HPVECs and found that the functions of DEGs were mainly associated with metabolic pathways, actin skeleton regulation, inflammatory pathways, and other disease-related pathways, which were highly consistent with functional experiments. The RNA-Seq results further proved the reliability of our research and showed that HPMSCs-Exo could be a promising therapeutic carrier. MiRNAs play an important role in the differentiation, proliferation, cytoskeleton, apoptosis, catabolism, and signaling pathway regulation of vascular endothelial cells (VECs) [40, 41]. Further exploration of miRNA expression levels of HPMSCs-Exo showed that there were several miRNAs, mainly including hsa-miR-148a-3p, hsa-miR-151a-3p, and hsa-miR-423-5p. The functions of these miRNAs affected cell adhesion, migration, angiogenesis, and apoptosis [42, 43]. After comparing the differential mRNAs of endothelial cells with the target genes of HPMSCs-Exo miRNA, we found that there were a variety of overlaps, and most of them were related to cytoskeletal function, which indicated that these miRNAs might play a role by co-regulating the changes of mRNA levels in endothelial cells after exosomes were cocultured with pulmonary VECs. Biological analysis shows that there are four binding sites between hsa-miR-148a-3p and ROCK1. The overexpression of hsa-miR-148a-3p was performed via transfecting HPMSCs with hsa-miR-148a-3p mimic. Compared with NC-mimic-HPMSCs-Exo, mimic-HPMSCs-Exo could further promote cell migration and angiogenesis. Phalloidin staining supported this result as well. Microscopically, the cell volume increased, and the cytoskeletal structure was further thickened. Immunoblot analysis showed that HPMSCs-Exo could further reduce the expression level of ROCK1 in HPVECs. But, the therapeutic effect in ROCK1-overexpressing cells was weakened. Collectively, HPMSCs-Exo can protect HPVECs by affecting the cytoskeleton, and the hsa-miR-148a-3p/ROCK1 pathway can play an important role, which is a potential treatment for ALI/ARDS.

## 5. Conclusions

In summary, HPMSCs-Exo may alleviate ALI/ARDS by improving the cytoskeleton and barrier function of HPVECs, where the hsa-miR-148a-3p/ROCK1 pathway may play an important role.

## Figures and Tables

**Table 1:** The primer sequences used for the q-PCR.

Gene name	Forward (5′−3′)	Reverse (5′−3′)
GAPDH	AATCCCATCACCATCTTCCA	TGGACTCCACGACGTACTCA
U6	CTCGCTTCGGCAGCACA	AACGCTTCACGAATTTGCGT
miR-148a-3p	TCAGTGCACTACAGAACTTTGT	GTCACCCCTGTTTCTGGCAC

## Data Availability

The datasets used during the present study are available from the corresponding author upon reasonable request.
